# Genome Sequences of Four Shigella boydii Strains Representative of the Major S. boydii Clades

**DOI:** 10.1128/MRA.00881-20

**Published:** 2020-10-08

**Authors:** Michael J. Sikorski, Tracy H. Hazen, Gopi Vyas, Jane M. Michalski, David A. Rasko

**Affiliations:** a Institute for Genome Sciences, University of Maryland School of Medicine, Baltimore, Maryland, USA; b Department of Microbiology and Immunology, University of Maryland School of Medicine, Baltimore, Maryland, USA; c Center for Vaccine Development and Global Health, University of Maryland School of Medicine, Baltimore, Maryland, USA; University of Rochester School of Medicine and Dentistry

## Abstract

There are four bacterial species in the genus *Shigella* that cause shigellosis or dysentery. Shigella boydii is one of the least studied *Shigella* species but has been shown to be separated into three phylogenomic clades. Here, we report four complete reference sequences of the S. boydii phylogenomic clades.

## ANNOUNCEMENT

Shigellosis is a diarrheal disease caused by the bacterial genus *Shigella*, consisting of four species (1), including Shigella boydii. The aim of this submission is to describe the complete reference genomes of four S. boydii isolates which are representatives of the previously identified S. boydii phylogenomic clades (2).

The four S. boydii strains were isolated using standard culture methods (3) from the stool of children in Bangladesh under the age of five as part of the Global Enteric Multicenter Study (GEMS) (3, 4). The isolates were grown in lysogeny broth overnight at 37°C, and DNA was purified by alkaline lysis extraction as previously described (5), with the exception that after the phenol:chloroform treatment the upper aqueous phase was added to a heavy phase lock tube (5 Prime, Inc., Gaithersburg, MD), and the extraction was repeated using chloroform-isoamyl alcohol (24:1 [vol/vol]). The upper aqueous phase was collected, and at least 5 volumes of isopropanol was used for precipitation of DNA on ice for 15 min, followed by centrifugation at 12,000 × *g* for 10 min, ethanol washes, and resuspension in water. Library preparation was conducted using a Kappa kit for 150-bp paired-end reads, and the genomes were sequenced on the Illumina HiSeq 2000 platform using paired-end libraries with 300-bp inserts, as previously described (2). The same genomic DNA preparations of each isolate were also used to generate a 20-kb sequencing library for the Pacific Biosciences (PacBio) RS II platform with P6C4 chemistry in a single flow cell per isolate using standard methods (6). The PacBio raw data of S. boydii isolates 600080, 600690, and 602068 were assembled using the Hierarchical Genome Assembly Process v.3 (HGAP3) pipeline and SMRTAnalysis v.2.3.0 software (7), while isolate 600657 was assembled using Canu v.1.4 (8). Assemblies for each isolate were circularized using Minimus2 (9) and polished with the generated Illumina reads using Quiver (7) to close the genomes. All software was run with default values. The total number of reads generated for each isolate on each sequencing platform and the relevant statistics for each genome assembly are listed in Table 1. The assemblies contained between two and four molecules, with each assembly having a single manually circularized chromosome and one or two circularized plasmids that ranged in size from 6.9 kb to 216 kb. Complete and circularized molecules were manually edited to remove overlaps and not rotated. The genomes were annotated with PGAP v.4.12 (12).

**TABLE 1:** Sequencing statistics

Isolate	E. coli/*Shigella* phylogroup	S. boydii clade	No. of contigs	Genome size (bp)	Total genome GC %	Molecule	Completion level	Molecule length (bp)	GC % by molecule	Plasmid incompatibility type	No. of Illumina reads	No. of PacBio reads	PacBio mean read length (bp)	Illumina sequence coverage (×)	PacBio sequence coverage (×)	*Shigella* virulence genes^*a*^	Antimicrobial resistance gene(s)^*b*^	Genome accession no.	SRA accession no.
600080	B1	1a	2	4,628,191	50.94	Chromosome	Circular	4,559,517	50.92	NA^*c*^	9,383,921	11,921	8,734	304.13	22.5	Aerobactin iron uptake (***iucABCD, iutA***), SHI-2, **SHI-3**	ND^*d*^	CP049606	SRX8156206, SRX8156207
						p600080_68	Circular	68,674	52.72	IncFIB(K)						None	*qnrS1*, *bla*_TEM-1_	CP049607	
600690	B1	1b	3	5,043,708	50.45	Chromosome	Circular	4,820,649	50.68	NA	6,758,931	16,566	7,813	201.01	25.7	Aerobactin iron uptake (***iucABCD, iutA)***, T6SS, SHI-2, SHI-3	*sat-2*	CP049278	SRX8156204, SRX8156205
						p600690_216	Circular	216,073	45.47	IncFII						***icsA*** (***virG***), ***icsP*** (***sopA***), ***mxi-ipa-spa***, **ShET2**,* SHI-3	ND	CP049279	
						p600690_6	Circular	6,986	49.00	None						None	ND	CP049280	
600657	B1	2	4	4,833,494	51.06	Chromosome	Circular	4,589,517	51.29	NA	3,611,833	15,166	8,621	112.09	27.1	Aerobactin iron uptake (***iucABCD, iutA***), ***sigA***, T2SS, SHI-2, SHI-3	*ampH*, *sat-2*	CP049281	SRX8163693, SRX8163694
						p600657_208	Circular	208,836	45.92	IncFII						***icsA*** (***virG)***, ***icsP*** (***sopA***), ***mxi-ipa-spa***, **ShET2**	ND	CP049282	
						p600657_26	Not circular	26,762	51.52	NA						None	ND	CP049283	
						p600657_8	Circular	8,379	55.48	None						None	*sul2*	CP049284	
602068	B1	3	2	4,758,031	50.92	Chromosome	Circular	4,572,715	51.15	NA	3,763,064	12,550	11,556	118.63	30.5	Aerobactin iron uptake (***iucABCD, iutA***), ***sigA***, ***T2SS***, SHI-2, SHI-3	*ampH*, *pmrF*, *sat-2*	CP049285	SRX8156202, SRX8156203
						p602068_185	Circular	185,316	45.12	IncFII						***icsA*** (***virG***), ***icsP (sopA)***, ***mxi-ipa-spa***, **ShET2**	ND	CP049286	

a Virulence genes listed in the Virulence Factor Database (10) where >50% of the genes of a particular region were detected with a BLAST score ratio (BSR) of >0.8 are reported. Bold type indicates that 100% of genes were detected with a BSR of >0.8.

b Antimicrobial resistance (AMR) genes were predicted using CARD RGI (11), and only “perfect” hits are reported here. Highly conserved genes associated with antibiotic resistance phenotypes, such as efflux pumps and select transcriptional regulators, are not included.

c NA, not applicable.

d ND, not detected.

**FIG 1:**
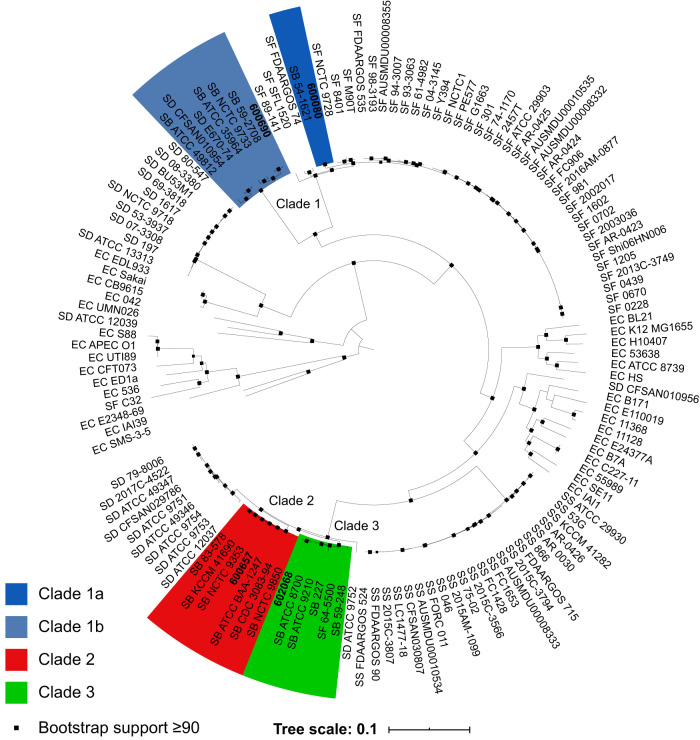
Phylogenomic tree containing four reference S. boydii isolates (in bold), reference *Shigella* isolates currently available in the public databases (*n* = 102), and a collection of diverse reference E. coli isolates (*n* = 30). Three S. boydii clades are identified by color, with clade 1 in blue (separated into two smaller subclades denoted by two shades of blue), clade 2 in red, and clade 3 in green. The tree was inferred with Interactive Tree Of Life (iTOL) v.5.5.1 with bootstrap support values greater than or equal to 90% shown at the black square nodes. Distance for the number of nucleotide changes is shown to be 0.1 with the corresponding tree scale bar length.

Phylogenomic analysis was performed on these four complete S. boydii genomes in comparison with complete *Shigella* genomes available from GenBank (*n* = 102) and a panel of diverse E. coli genomes (*n* = 30) as previously described (2). A maximum-likelihood phylogeny tree (Fig. 1) was constructed with 100 bootstrap replicates using RAxML v.8.2.10 (13) and visualized using iTOL v.5.5.1 (10).

The presence or absence of *Shigella* virulence genes (14) was examined using the large-scale BLAST score ratio (11) (Table 1). Antibiotic resistance genes were identified in each assembly using the Resistance Gene Identifier (RGI) v.5.1.0 software against the Comprehensive Antibiotic Resistance Database (CARD) v.3.0.8 with perfect detection criteria (15), and plasmid incompatibility types were predicted using PlasmidFinder (Table 1) (16). Three of the assemblies (600690, 600657, and 602068) contained the *Shigella* virulence plasmid, while the fourth assembly (600080) lacked the *Shigella* virulence plasmid but contained a 68-kb plasmid with the predicted antibiotic resistance genes *qnrS1* and *bla*_TEM-1_ (Table 1).

Given the paucity of S. boydii reference isolates, these four complete phylogenomically distinct genomes presented may serve future studies as representative references from each clade of S. boydii (2).

### Data availability.

All data have been released, and the accession numbers are listed in Table 1.
